# *Clostridioides difficile* Infection in Hospitalised COVID-19 Patients: Antibiotic Exposure, Cardiovascular Comorbidities, and Clinical Outcomes in a Romanian Tertiary Infectious Diseases Centre

**DOI:** 10.3390/jcm15145488

**Published:** 2026-07-13

**Authors:** Cristiana Georgeta Bujor, Marilena Dinuti, Felicia Sfrijan, Alina Ramona Buzatu

**Affiliations:** Department IV—Biochemistry and Pharmacology, Faculty of Medicine, “Victor Babeş” University of Medicine and Pharmacy Timișoara, Eftimie Murgu Square 2, 300041 Timișoara, Romania; bujor.cristiana@umft.ro (C.G.B.); buzatu.ramona@umft.ro (A.R.B.)

**Keywords:** *Clostridioides difficile*, COVID-19, SARS-CoV-2, heart failure, antibiotic stewardship, nosocomial infection, piperacillin/tazobactam, sepsis, logistic regression

## Abstract

**Background/Objectives**: *Clostridioides difficile* infection (CDI) is an increasingly recognised nosocomial complication in patients hospitalised with coronavirus disease 2019 (COVID-19), driven by broad-spectrum antibiotic exposure, corticosteroid use, and severe acute respiratory syndrome coronavirus 2 (SARS-CoV-2)-related intestinal dysbiosis. The contribution of underlying cardiovascular comorbidities, particularly heart failure, to CDI susceptibility in hospitalised COVID-19 patients remains insufficiently characterised. We aimed to evaluate the prevalence, risk factors, and clinical impact of CDI in hospitalised COVID-19 patients at a tertiary Romanian infectious diseases centre, with a specific focus on cardiovascular disease and modifiable antimicrobial-prescribing factors. **Methods**: This single-centre retrospective observational cohort study enrolled 395 consecutive adult patients hospitalised with SARS-CoV-2 infection confirmed by reverse transcription polymerase chain reaction (RT-PCR) at the “Victor Babeș” Clinical Hospital for Infectious Diseases and Pneumophthisiology, Timișoara, Romania, between 1 March 2020 and 31 December 2024. Patients were stratified into a CDI group (*n* = 24) and a non-CDI group (*n* = 371) on the basis of hospital-onset CDI. Demographic, clinical, laboratory, therapeutic, and outcome variables were extracted from electronic medical records. Continuous variables were compared using Student’s independent-samples *t*-test, categorical variables using Fisher’s exact test; odds ratios (ORs) with exact 95% confidence intervals (CIs) were computed for all binary comparisons, and a parsimonious multivariable logistic regression was used to adjust the antibiotic-exposure effect for age. Temporal relationships between CDI onset and sepsis documentation were also analysed. A two-sided *p*-value < 0.05 was considered statistically significant. **Results**: CDI was identified in 24 patients (prevalence 6.1%). The CDI and non-CDI groups were comparable in age (71.1 ± 10.2 vs. 71.7 ± 11.4 years; *p* = 0.817), sex distribution (50.0% vs. 45.8% female; *p* = 0.833), and vaccination status (*p* = 0.383). Heart failure prevalence did not differ between groups (29.2% vs. 26.7%; OR 1.13, 95% CI 0.46–2.81; *p* = 0.813). Any antibiotic therapy was strongly associated with CDI (95.8% vs. 70.6%; OR 9.57, 95% CI 1.28–71.74; *p* = 0.004), as was longer duration of antibiotic therapy (8.1 ± 2.2 vs. 5.2 ± 3.7 days; *p* < 0.001). In the multivariable model, each additional day of antibiotic therapy was independently associated with a 13.6% increase in the odds of CDI (adjusted OR 1.14 per day, 95% CI 1.03–1.26; *p* = 0.013) after adjustment for age. Piperacillin/tazobactam accounted for 65.2% (15/23) of the recorded pre-CDI broad-spectrum antibiotic regimens among antibiotic-exposed CDI patients. Documented sepsis was substantially more frequent in CDI patients (95.8% vs. 15.4%; *p* < 0.001) and length of hospitalisation was prolonged (15.6 ± 8.2 vs. 12.0 ± 8.1 days; *p* = 0.038). In-hospital mortality did not differ significantly (4.2% vs. 7.5%; *p* = 1.000). **Conclusions**: Hospital-onset CDI complicated 6.1% of hospitalised COVID-19 admissions and was independently associated with the cumulative duration of antibiotic therapy. Heart failure was not significantly associated with CDI in this cohort; however, given the limited number of CDI events, this should be interpreted as absence of evidence rather than evidence of absence. CDI was also associated with prolonged hospitalisation and high co-occurrence of documented sepsis, although causality cannot be inferred from the retrospective design. These findings support strengthened antibiotic stewardship and targeted CDI surveillance in COVID-19 inpatient wards.

## 1. Introduction

*Clostridioides difficile* (formerly *Clostridium difficile*) is a Gram-positive, spore-forming anaerobic bacillus responsible for a spectrum of healthcare-associated gastrointestinal disease ranging from mild antibiotic-associated diarrhoea to fulminant pseudomembranous colitis, toxic megacolon, and death [1,2,3]. Disruption of the commensal gut microbiota by systemic antibiotics is the principal predisposing factor, allowing germination of ingested *C. difficile* spores and toxin-mediated colonic injury [4,5,6,7]. Established CDI risk factors include antibiotic exposure, advanced age, comorbidity burden, prolonged hospitalisation, and additional pharmacological exposures [6,8]. During the COVID-19 pandemic, empirical antibiotic prescribing was frequently disproportionate to the documented prevalence of bacterial coinfection, creating a favourable context for antimicrobial-pressure-driven healthcare-associated complications, including CDI [9,10,11].

Since the emergence of SARS-CoV-2 in 2020, the epidemiology of CDI has shifted under the combined pressure of widespread broad-spectrum empirical antibiotic prescribing for suspected bacterial superinfection, near-universal use of systemic corticosteroids in moderate-to-severe COVID-19 [12], and direct viral injury to the gastrointestinal tract through angiotensin-converting enzyme 2 (ACE2)-mediated enterocyte invasion [13]. Substantial alterations of the gut microbiome have been documented in COVID-19, with reduced microbial diversity and depletion of butyrate-producing taxa such as *Faecalibacterium prausnitzii*—changes that biologically lower colonisation resistance against *C. difficile* [14,15].

Several international cohorts have reported variable CDI rates among hospitalised COVID-19 patients, ranging from approximately 1% to 10% [16,17,18,19]. Cardiovascular disease—and heart failure in particular—is among the most prevalent comorbidities in hospitalised COVID-19 patients and could theoretically increase CDI susceptibility through intestinal hypoperfusion, venous congestion, and microbiome alterations linked to chronic cardiac dysfunction [20,21,22]. However, the specific contribution of heart failure to CDI risk in COVID-19 inpatients has not been clearly delineated, and data from Romania and Eastern Europe remain sparse. Moreover, because patient profiles and the prognostic weight of individual predictors have been shown to shift substantially across successive pandemic waves [23], findings from any single observation period must be interpreted in the light of this temporal heterogeneity.

The objectives of the present study were therefore to: (i) determine the prevalence of hospital-onset CDI among adult patients hospitalised with COVID-19 at a tertiary infectious diseases centre in western Romania; (ii) evaluate demographic, cardiovascular, clinical, and therapeutic factors associated with CDI occurrence, including a parsimonious adjusted analysis of antibiotic exposure; and (iii) assess the clinical impact of CDI on sepsis documentation, length of hospitalisation, intensive care unit (ICU) admission, and in-hospital mortality. Particular attention was given to cardiovascular comorbidities—especially heart failure—and to antibiotic exposure as a potentially modifiable risk factor.

## 2. Materials and Methods

### 2.1. Study Design and Setting

We conducted a single-centre retrospective observational cohort study at the “Victor Babeș” Clinical Hospital for Infectious Diseases and Pneumophthisiology in Timișoara, Romania—a tertiary regional referral centre for infectious diseases and pulmonology serving the western part of the country. The study period extended from 1 March 2020 to 31 December 2024, encompassing all major SARS-CoV-2 pandemic waves (ancestral/Alpha, Delta, and Omicron variants) and the early post-acute pandemic phase. The study was reported in accordance with the Strengthening the Reporting of Observational Studies in Epidemiology (STROBE) statement for observational studies [24].

### 2.2. Study Population

Inclusion criteria were: (i) age ≥ 18 years at admission; (ii) laboratory-confirmed SARS-CoV-2 infection by real-time reverse-transcription polymerase chain reaction (RT-PCR) from a nasopharyngeal swab; (iii) hospitalisation for COVID-19-related illness for at least 24 h; and (iv) availability of complete medical and laboratory records. Exclusion criteria were: (i) documented CDI present at admission (i.e., community-onset CDI); (ii) any documented history of CDI within the 12 weeks preceding the index admission; (iii) transfer from another hospital with ≥7 days of prior antibiotic exposure; and (iv) incomplete clinical or laboratory data preventing classification.

### 2.3. Case Definitions

CDI was defined in accordance with European Society of Clinical Microbiology and Infectious Diseases (ESCMID) diagnostic guidance as the occurrence of ≥3 unformed stools within 24 h together with laboratory evidence of toxigenic C. difficile, in the absence of an alternative explanation [25]. Only hospital-onset CDI episodes occurring > 48 h after admission were included. Patients with documented CDI at admission or suspected community-onset CDI were excluded.

Sepsis was recorded when Sepsis-3 criteria [26] were documented during the hospital course. Sepsis was analysed as a clinical outcome occurring during hospitalisation and was not assumed to be directly attributable to CDI in all cases, given the frequent coexistence of severe COVID-19, bacterial superinfection, and other nosocomial complications. To address the directionality of this association, we additionally analysed the temporal relationship between CDI onset and the first documented sepsis episode in CDI patients (see Section 2.6).

### 2.4. Data Collection

Data were extracted from the hospital’s electronic medical record system using a structured, predefined data collection form. Variables collected included: demographics (age, sex, urban/rural residence, body mass index [BMI]); COVID-19 vaccination status (number of doses and vaccine type); cardiovascular comorbidities (heart failure with New York Heart Association (NYHA) class, ischaemic heart disease, atrial fibrillation, prior stroke, grade of hypertension); non-cardiovascular comorbidities (type 1 and type 2 diabetes mellitus, chronic obstructive pulmonary disease (COPD), chronic kidney disease, hepatic disease, neurological disease, malignancies); inflammatory and coagulation biomarkers at admission and discharge; COVID-19 treatments (remdesivir, favipiravir, anakinra, systemic corticosteroids, and antibiotics—agent and duration); oxygen therapy details; ICU admission; and clinical outcomes (sepsis, septic shock, length of stay, in-hospital death).

For patients diagnosed with CDI, treatment data were additionally collected, including the use of oral vancomycin, fidaxomicin, or metronidazole; recurrence during the same admission when available; and CDI-related complications such as toxic megacolon, colectomy, or ICU escalation. CDI recurrence was ascertained only within the index hospitalisation; post-discharge follow-up and out-of-hospital recurrence were not systematically recorded in the electronic medical record and were therefore not analysed.

### 2.5. Laboratory and Imaging Analyses

All laboratory analyses were performed in the Department of Laboratory Medicine of the “Victor Babeș” Clinical Hospital for Infectious Diseases and Pneumophthisiology, Timișoara, in accordance with the local ISO 15189 quality framework. Microbiological identification and antimicrobial susceptibility testing were interpreted using current European Committee on Antimicrobial Susceptibility Testing (EUCAST) clinical breakpoints. The instruments and methods used for each category of analysis are summarised in Table 1. Detailed organism-level blood-culture and antimicrobial-susceptibility data were systematically abstracted for the CDI subgroup. Comparable organism-level microbiological data were not uniformly available for the non-CDI group; therefore, these findings were analysed descriptively and no between-group comparison of bloodstream pathogens or resistance phenotypes was performed.

Real-time RT-PCR for SARS-CoV-2 detection was performed on nasopharyngeal swabs collected in viral transport medium, processed using CE-IVD-marked extraction kits and amplification panels targeting the E, N, and ORF1ab genes. Chest imaging (X-ray and multi-slice computed tomography) was acquired and reported on hospital digital radiology platforms by board-certified radiologists.

### 2.6. Statistical Analysis

Continuous variables were expressed as mean ± standard deviation (SD) and compared between groups using Student’s independent-samples *t*-test after verifying the assumption of approximate normality with the Shapiro–Wilk test. For variables with skewed distribution, the Mann–Whitney U test was used as a sensitivity analysis. Categorical variables were expressed as absolute frequencies and percentages and compared using Fisher’s exact test, given the small number of CDI events. Odds ratios (ORs) with exact 95% confidence intervals (CIs) were calculated for all binary comparisons. The Haldane–Anscombe continuity correction (addition of 0.5 to all cells) was applied for 2 × 2 tables containing a zero cell.

Because the number of CDI events was limited (*n* = 24), we deliberately restricted the multivariable analysis to a parsimonious logistic regression model with no more than two covariates, in order to respect the conventional events-per-variable rule (≥10) and avoid model overfitting. The model included CDI occurrence as the dependent variable and (i) the cumulative duration of antibiotic therapy in days and (ii) patient age in years as covariates. Age was retained as a clinically relevant adjustment variable on the basis of its well-established association with CDI in published literature. Adjusted ORs are reported per one-day increase in antibiotic duration and per one-year increase in age.

To address the directionality of the strong observed association between CDI and documented sepsis, we extracted the calendar date of CDI laboratory confirmation and the calendar date of the first sepsis episode for each CDI patient, and classified the temporal sequence as: sepsis preceding CDI (>24 h before CDI confirmation), sepsis concurrent with CDI (±24 h), or sepsis following CDI (>24 h after CDI confirmation).

A two-sided *p*-value < 0.05 was considered statistically significant. All associations from univariable analyses should be interpreted as unadjusted, exploratory estimates; only the antibiotic-duration effect from the multivariable model is reported as an adjusted association. All analyses were performed using Python 3.12 with SciPy 1.11, pandas 2.0, and statsmodels 0.14.

### 2.7. Ethical Considerations

Patient data were collected at the time of hospitalisation, and written informed consent for the use of their anonymised clinical data for research purposes was obtained from all participants at admission. The retrospective analysis of these data for the present study was subsequently approved by the Ethics Committee of the “Victor Babeș” Clinical Hospital for Infectious Diseases and Pneumophthisiology, Timișoara (approval no. 4711/20 May 2026), explicitly covering the study period 1 March 2020–31 December 2024. The study was conducted in accordance with the principles of the Declaration of Helsinki and the EU General Data Protection Regulation (Regulation (EU) 2016/679). All data were anonymised prior to analysis, and the study was conducted in accordance with institutional ethical requirements and GDPR regulations.

## 3. Results

### 3.1. Study Population and CDI Prevalence

A total of 395 adult patients with RT-PCR-confirmed SARS-CoV-2 infection met the inclusion criteria and were included in the final analysis. CDI was identified in 24 patients (6.1%), while 371 patients (93.9%) had no CDI during their hospital admission. The overall clinical profile was that of elderly multimorbid patients with a high prevalence of cardiovascular and metabolic comorbidities, in line with European hospitalised-COVID-19 cohorts. All CDI cases fulfilled the predefined criteria for hospital-onset CDI, occurring more than 48 h after admission.

### 3.2. Demographic and Clinical Characteristics

**Figure 1:**
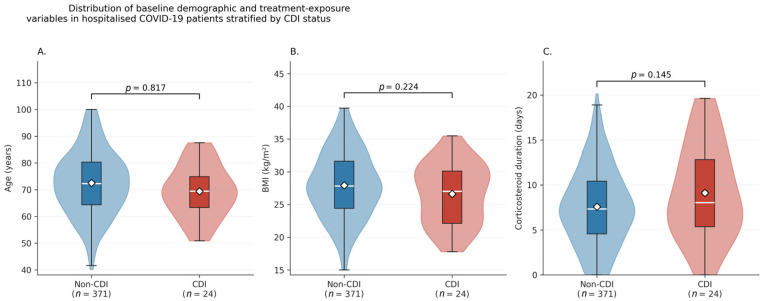
Violin plots showing the full distribution of (**A**) age at admission, (**B**) body mass index, and (**C**) duration of corticosteroid exposure in hospitalised COVID-19 patients stratified by CDI status. The internal box plot indicates median (white horizontal line), interquartile range (filled box), and 1.5 × IQR whiskers; the white diamond marker indicates the arithmetic mean. Reported *p*-values correspond to Student’s independent-samples *t*-test.

Demographic and baseline clinical characteristics were comparable between groups. The mean age was 71.1 ± 10.2 years in the CDI group versus 71.7 ± 11.4 years in the non-CDI group (*p* = 0.817). Female sex was present in 50.0% of CDI patients and 45.8% of non-CDI patients (*p* = 0.833). The mean BMI was 26.8 ± 4.9 kg/m^2^ versus 28.1 ± 5.2 kg/m^2^, respectively (*p* = 0.224). COVID-19 vaccination coverage was 58.3% in the CDI group versus 66.8% in the non-CDI group (*p* = 0.383). The distributions of age, BMI, and corticosteroid exposure duration are shown in Figure 1.

**Figure 2:**
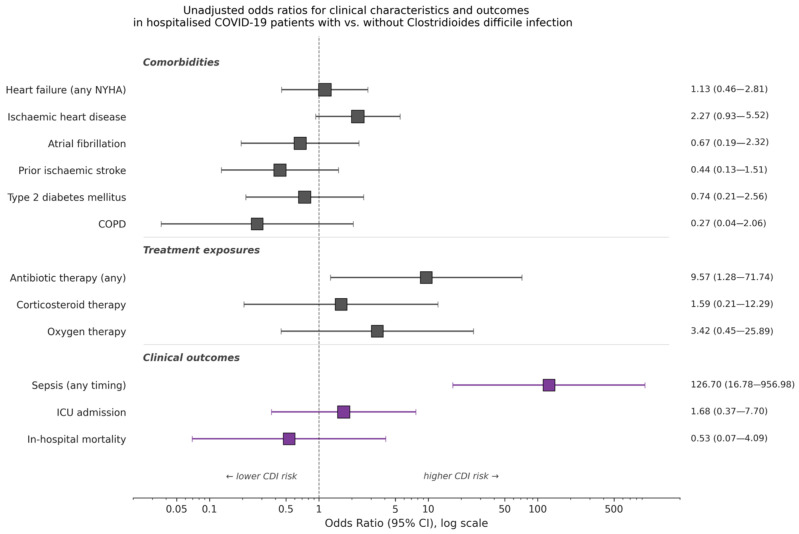
Forest plot of unadjusted odds ratios with 95% confidence intervals for the association between baseline comorbidities, treatment exposures, and clinical outcomes and the occurrence of hospital-onset *Clostridioides difficile* infection (CDI) in hospitalised COVID-19 patients (*n* = 395). The vertical dashed line marks the null effect (OR = 1). Square markers represent point estimates; horizontal lines represent 95% confidence intervals. Marker size is proportional to the precision of the estimate (inverse of the log CI width). Variables in violet correspond to clinical outcomes occurring during hospitalisation.

Regarding cardiovascular comorbidities, heart failure of any NYHA class was present in 29.2% of CDI patients versus 26.7% of non-CDI patients (OR 1.13, 95% CI 0.46–2.81; *p* = 0.813). Ischaemic heart disease showed a higher numerical prevalence in CDI patients (33.3% vs. 18.1%; OR 2.27, 95% CI 0.93–5.52; *p* = 0.101) but did not reach statistical significance. Atrial fibrillation (12.5% vs. 17.5%; OR 0.67, 95% CI 0.19–2.32; *p* = 0.780) and prior ischaemic stroke (12.5% vs. 24.5%; OR 0.44, 95% CI 0.13–1.51; *p* = 0.222) did not differ significantly. Notably, no CDI patient had documented chronic kidney disease, compared with 9.2% in the non-CDI group. Complete demographic, clinical, therapeutic, and outcome data with ORs and 95% CIs are presented in Table 2, and the corresponding forest plot is shown in Figure 2.

### 3.3. Antibiotic Exposure and Treatment Profiles

**Figure 3:**
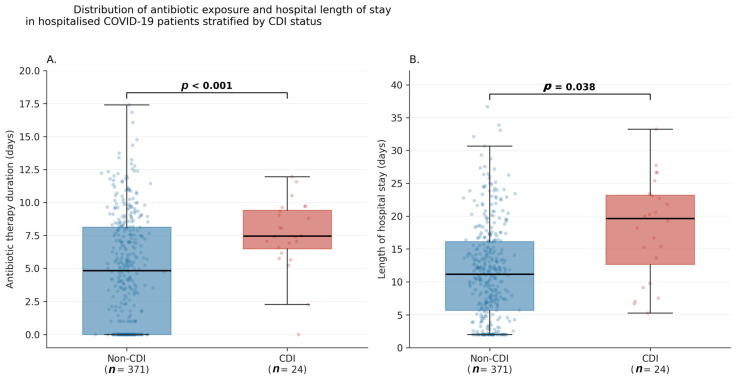
Distribution of (**A**) duration of antibiotic therapy and (**B**) length of hospital stay in hospitalised COVID-19 patients stratified by CDI status. Box plots show median (thick horizontal line), interquartile range (box), and 1.5 × IQR whiskers; individual patient values are overlaid as jittered points. Reported *p*-values correspond to Student’s independent-samples *t*-test.

Antibiotic therapy was administered to 23 of 24 patients in the CDI group (95.8%), compared with 262 of 371 patients in the non-CDI group (70.6%; OR 9.57, 95% CI 1.28–71.74; *p* = 0.004). The mean duration of antibiotic therapy was significantly longer in the CDI group than in the non-CDI group (8.1 ± 2.2 vs. 5.2 ± 3.7 days; *p* < 0.001) (Figure 3A). After adjustment for age in a parsimonious multivariable logistic regression, each additional day of antibiotic therapy was independently associated with a 13.6% increase in the odds of CDI (adjusted OR 1.14 per day, 95% CI 1.03–1.26; *p* = 0.013), whereas age was not independently associated with CDI in this model (adjusted OR 0.99 per year, 95% CI 0.96–1.03; *p* = 0.630).

It is important to clarify that the antibiotic regimens summarised in Table 3 refer to the broad-spectrum systemic antibiotics administered to CDI patients before the onset of hospital-onset CDI, for the treatment of suspected or documented COVID-19-associated bacterial superinfection; they represent the antecedent antibiotic exposure that constitutes the principal modifiable risk factor for CDI and are not, in any case, the antibiotics used to treat CDI itself. These broad-spectrum agents were initiated empirically on clinical and inflammatory grounds (fever, rising procalcitonin/C-reactive protein, radiological progression) and, where blood cultures were obtained, were subsequently adjusted according to culture and susceptibility results (Section 3.4). Among the 23 CDI patients who had received systemic antibiotics before CDI onset, piperacillin/tazobactam was the most frequently prescribed broad-spectrum regimen, accounting for 65.2% of pre-CDI antibiotic exposures, followed by carbapenem-based regimens (Table 3). Once CDI was diagnosed—on the basis of new-onset clinical diarrhoea (≥3 unformed stools within 24 h) and a positive two-step laboratory algorithm—all 24 CDI patients were treated with oral vancomycin at a dose of 125 mg four times daily for 10 days, in accordance with current ESCMID and IDSA/SHEA guidance [27,28]. CDI-directed therapy was initiated at the time of clinical suspicion and continued after laboratory confirmation. No patient received fidaxomicin or metronidazole as first-line CDI therapy. Where clinically feasible, the antecedent broad-spectrum systemic antibiotic regimen was discontinued or de-escalated at the time of CDI diagnosis as part of antimicrobial stewardship. Detailed regimen-level antecedent antibiotic exposure was systematically recorded in the CDI group but not in all non-CDI patients; consequently, regimen-specific risk comparisons between groups were not possible, and the distribution in Table 3 is presented as descriptive rather than as evidence of regimen-specific causal risk. Systemic corticosteroid therapy was administered with similar frequency in both groups (95.8% vs. 93.5%; *p* = 1.000) and for similar durations (8.9 ± 3.8 vs. 7.5 ± 4.6 days; *p* = 0.145). The need for supplemental oxygen therapy was high in both groups, but did not differ significantly (95.8% vs. 87.1%; *p* = 0.337).

### 3.4. Microbiological Findings in the CDI Group: Blood Cultures, Pathogen Identification, and Antimicrobial Susceptibility

At least one set of peripheral blood cultures was obtained during the septic work-up in 22 of the 24 CDI patients (91.7%); the two remaining patients had no blood cultures drawn before empirical antibiotic adjustment. Blood cultures yielded a clinically significant pathogen in 9 of these 22 patients (40.9%), were sterile in 12 (54.5%), and grew a probable skin-contaminant in 1 (4.5%). Among the 9 patients with clinically significant bacteraemia, the predominant isolates were Gram-negative enteric organisms—*Escherichia coli* (*n* = 3) and *Klebsiella pneumoniae* (*n* = 3)—followed by *Enterococcus faecium* (*n* = 2) and *Pseudomonas aeruginosa* (*n* = 1). The single contaminant isolate was a coagulase-negative *Staphylococcus epidermidis* recovered from a single bottle, judged to be a contaminant on clinical and microbiological grounds and not treated as a true bloodstream pathogen. Species identification was confirmed by MALDI-TOF mass spectrometry, and antimicrobial susceptibility testing was performed on the VITEK 2 Compact system with interpretation according to current EUCAST clinical breakpoints. The predominance of gut-derived Gram-negative and enterococcal organisms among bloodstream isolates is consistent with the antibiotic-exposed, dysbiotic intestinal milieu that also predisposes to CDI.

With respect to antimicrobial susceptibility, the three *E. coli* isolates were susceptible to carbapenems and piperacillin/tazobactam; one of the three was an extended-spectrum β-lactamase (ESBL)-producing strain resistant to third-generation cephalosporins. Of the three *K. pneumoniae* isolates, two were ESBL-producing and one was carbapenem-susceptible without an ESBL phenotype; no carbapenemase-producing isolates were identified in this CDI subgroup. Both *E. faecium* isolates were susceptible to vancomycin and linezolid (no vancomycin-resistant enterococci were detected), and the single *P. aeruginosa* isolate was susceptible to meropenem and ceftazidime. Importantly, the bloodstream isolates and their susceptibility profiles relate to the patients’ concurrent or antecedent bacterial sepsis and bacteraemia, not to *C. difficile*, which is an anaerobic enteric pathogen diagnosed by the stool two-step algorithm rather than by blood culture and was treated with oral vancomycin (Section 3.3). Sepsis (Sepsis-3 criteria) was therefore microbiologically confirmed by a positive blood culture in 9 of the 23 CDI patients with documented sepsis (39.1%), whereas in the remaining 14 patients, sepsis was a clinical-laboratory diagnosis (organ dysfunction with documented or strongly suspected infection) without a positive blood culture, in keeping with the high rate of prior empirical antibiotic exposure that reduces blood-culture yield. The full distribution of blood-culture results, identified pathogens, and key resistance phenotypes is summarised in Table 4.

### 3.5. Clinical Outcomes and Temporal Relationship Between CDI and Sepsis

Documented sepsis occurred in 23 of 24 CDI patients (95.8%) versus 57 of 371 non-CDI patients (15.4%; OR 126.70, 95% CI 16.78–956.98; *p* < 0.001). To clarify the directionality of this association, we examined the temporal sequence of CDI laboratory confirmation and the first documented sepsis episode in CDI patients: sepsis was documented before CDI confirmation in 17 of 23 CDI patients with sepsis (73.9%), concurrently (±24 h) in 4 patients (17.4%), and after CDI confirmation in 2 patients (8.7%). In the majority of cases, therefore, the documented septic state preceded the laboratory diagnosis of CDI and likely reflects systemic inflammation from severe COVID-19 and/or bacterial superinfection rather than *C. difficile* bacteraemia or translocation per se. CDI is therefore best interpreted in this cohort as a marker of an already inflammatory and antibiotic-exposed clinical trajectory, with potential synergistic contribution to deterioration.

Septic shock was rare and did not differ significantly between groups (0.0% vs. 1.6%; *p* = 1.000). ICU admission was numerically higher in the CDI group but did not reach statistical significance (8.3% vs. 5.1%; OR 1.68, 95% CI 0.37–7.70; *p* = 0.370). The length of hospital stay was significantly longer in CDI patients (15.6 ± 8.2 vs. 12.0 ± 8.1 days; *p* = 0.038) (Figure 3B). In-hospital mortality was numerically lower in CDI patients (4.2% vs. 7.5%; OR 0.53, 95% CI 0.07–4.09; *p* = 1.000), although this comparison was constrained by the small number of events in the CDI group. During the index hospitalisation, no CDI recurrence, toxic megacolon, colectomy, or CDI-related escalation to intensive care was documented.

### 3.6. Baseline Demographic and Treatment-Exposure Distributions

To complement the tabular and forest-plot summaries, the distributions of age, BMI, and corticosteroid exposure duration in the two groups are shown in Figure 1. The two groups overlapped substantially across all three variables, consistent with the absence of significant differences detected at the group-mean level.

## 4. Discussion

The present retrospective cohort study, conducted at a tertiary Romanian infectious diseases centre, identified a hospital-onset CDI prevalence of 6.1% among 395 hospitalised COVID-19 patients. This rate is consistent with the upper range of estimates reported in international cohorts, which span approximately 1–10% [16,17,18,19], and reflects the high antibiotic-prescribing intensity that characterised inpatient COVID-19 management during the pandemic in our setting. A previous analysis from the same institution focusing on antibiotic prescribing patterns showed that more than 88% of hospitalised COVID-19 patients received systemic antibiotics, with extensive use of World Health Organization (WHO) Watch/Reserve agents and an associated burden of secondary multidrug-resistant infections [29], providing a coherent epidemiological background for the present findings.

Contrary to our initial hypothesis, heart failure was not a statistically significant risk factor for CDI in this cohort (OR 1.13, 95% CI 0.46–2.81; *p* = 0.813), despite biologically plausible mechanisms—intestinal hypoperfusion, venous congestion, microbiome dysbiosis, and immune dysregulation—that link chronic cardiac dysfunction to impaired colonisation resistance [20,21,22]. A likely explanation is that, in a population uniformly exposed to broad-spectrum antibiotics, corticosteroids, and severe systemic inflammation, the pharmacological and inflammatory drivers of CDI dominate, potentially masking any independent contribution of heart failure. The numerically higher prevalence of ischaemic heart disease in CDI patients (33.3% vs. 18.1%; OR 2.27, 95% CI 0.93–5.52; *p* = 0.101) is suggestive but does not reach statistical significance and would require validation in larger, multicentre studies. Given the limited number of CDI events, the absence of a statistically significant heart-failure–CDI association should be interpreted as absence of evidence at this sample size rather than evidence of absence. It must be emphasised that, with only 24 CDI events and a heart-failure prevalence of approximately 27% in both groups, the study had very limited power to detect a clinically meaningful association: the 95% confidence interval around the heart-failure odds ratio (0.46–2.81) is wide and remains compatible with both a moderate protective effect and a near-threefold increase in risk. This non-significant result therefore cannot exclude a true association and should be read strictly as absence of evidence at the present sample size rather than as evidence of absence. Adequately powered, multicentre studies—ideally with formal adjustment for cumulative antibiotic exposure and illness severity—are required before any firm conclusion about the heart-failure–CDI relationship can be drawn.

We did not observe a significant difference in CDI occurrence by sex (female sex 50.0% in CDI vs. 45.8% in non-CDI patients; *p* = 0.833), and the modest event count precluded a dedicated sex-stratified analysis of outcomes. This null finding should nonetheless be interpreted against a substantial body of evidence documenting biologically grounded sex-related differences in COVID-19—including differences in innate and adaptive immune and inflammatory responses, hospitalisation patterns, and mortality—that have been shown to persist across pandemic waves [30]. The absence of a detectable sex effect on CDI in our cohort may therefore reflect genuine convergence of CDI risk once patients are uniformly exposed to broad-spectrum antibiotics, corticosteroids, and severe systemic inflammation, as much as limited statistical power, rather than a true absence of sex-related modulation. Sex-disaggregated reporting of healthcare-associated infections in COVID-19 inpatients remains warranted in larger cohorts.

The strongest and most clinically actionable association identified in this study was between cumulative antibiotic exposure and CDI. CDI patients had nearly tenfold higher unadjusted odds of having received antibiotics (OR 9.57, 95% CI 1.28–71.74; *p* = 0.004), and the mean duration of antibiotic therapy was substantially longer (8.1 vs. 5.2 days; *p* < 0.001). Importantly, in the parsimonious multivariable logistic regression adjusted for age, each additional day of antibiotic therapy was independently associated with a 13.6% increase in the odds of CDI (adjusted OR 1.14 per day, 95% CI 1.03–1.26; *p* = 0.013), confirming that the exposure–CDI association is not explained by simple confounding by age. We deliberately restricted the multivariable model to two covariates because of the limited number of CDI events (*n* = 24), in order to respect the events-per-variable rule (≥10) and avoid the overfitting that would result from a more elaborate model in this sample. Piperacillin/tazobactam accounted for 65.2% (15/23) of the recorded pre-CDI broad-spectrum antibiotic regimens among antibiotic-exposed CDI patients and belongs to the broad-spectrum exposures repeatedly associated with hospital-acquired CDI risk [7]. While our descriptive data are consistent with this pattern, the absence of comparable regimen-level data in the non-CDI group prevents formal regimen-specific risk inference. These findings reinforce the need for hospital-based antibiotic stewardship, particularly because antibiotic prescribing in COVID-19 has frequently exceeded the documented prevalence of bacterial coinfection [9,10,29].

A complementary mechanistic explanation derives from the marked alterations of the gut microbiome observed in COVID-19. A recent systematic review and meta-analysis demonstrated a consistent reduction in gut microbial alpha-diversity (pooled Shannon SMD = −0.69), with depletion of butyrate-producing taxa such as *Faecalibacterium prausnitzii* (pooled log-fold change = −1.24) and enrichment of opportunistic genera including *Enterococcus* spp. (pooled log-fold change = +1.45) in patients with SARS-CoV-2 infection [15]. This SARS-CoV-2–induced dysbiosis, compounded by antibiotic-driven disruption of the colonic microbiota, plausibly creates a permissive intraluminal environment for *C. difficile* spore germination and toxin production, supporting the high CDI rates documented during the pandemic [14,31].

The very high rate of documented sepsis observed in the CDI group (95.8%; OR 126.70 vs. non-CDI) is striking and must be interpreted with attention to operational definitions and temporality. In our dataset, “sepsis” encompasses any documented systemic inflammatory response with organ dysfunction during the hospital course meeting Sepsis-3 criteria, and is not necessarily attributable solely to *C. difficile* bacteraemia or translocation. The temporal analysis we performed (Section 3.5) is critical to interpretation: sepsis preceded the laboratory confirmation of CDI in approximately three-quarters of CDI patients with sepsis, indicating that CDI more often occurred in the context of an already established systemic inflammatory and antibiotic-exposed state rather than as the primary cause of sepsis. Furthermore, the extreme magnitude of the unadjusted OR (126.70) is, in our view, partially driven by immortal time bias: CDI patients had a longer length of stay (15.6 vs. 12.0 days), and thus greater exposure time during which sepsis could be diagnosed and documented. We have therefore interpreted this association conservatively and have not attempted to derive a causal effect of CDI on sepsis from this dataset. Even so, the very strong co-occurrence highlights that CDI in COVID-19 inpatients identifies a particularly vulnerable phenotype. This interpretation is supported by recent institutional data from western Romania showing that, among 395 patients hospitalised during the Omicron BA.5 wave, vaccination remained independently protective against in-hospital mortality, admission SpO_2_ and IL-6 were key mortality discriminators, and bloodstream bacterial co-infections were frequently dominated by gut-derived organisms [32,33].

The microbiological data reported in Section 3.4 reinforce this interpretation. Blood cultures were obtained in the great majority of CDI patients (91.7%) and yielded a clinically significant pathogen in 40.9% of cultured patients, so sepsis was microbiologically confirmed by bacteraemia in 39.1% of CDI patients with documented sepsis, while the remainder represented clinical-laboratory Sepsis-3 diagnoses without a positive blood culture—a pattern expected in a cohort with heavy prior empirical antibiotic exposure, which substantially lowers blood-culture yield. Crucially, the bloodstream isolates were dominated by gut-derived Gram-negative organisms (*Escherichia coli*, *Klebsiella pneumoniae*) and enterococci (*Enterococcus faecium*), with a minority of extended-spectrum β-lactamase-producing Enterobacterales and no carbapenemase-producing or vancomycin-resistant isolates detected in this subgroup. The predominance of enteric Gram-negative organisms and enterococci is compatible with severe illness, broad-spectrum antibiotic exposure, and intestinal dysbiosis. However, the primary source of bacteraemia was not systematically adjudicated, and gut translocation cannot be directly inferred from the present data. Consistent with guideline-concordant practice, all 24 CDI patients were treated with oral vancomycin, whereas the antecedent broad-spectrum regimens listed in Table 3—most frequently piperacillin/tazobactam—represent systemic antibiotic exposure preceding CDI rather than CDI-directed therapy.

The significant prolongation of hospital stay in CDI patients (15.6 vs. 12.0 days; *p* = 0.038) has direct implications for hospital resource utilisation, infection control, and patient experience, and is consistent with the international literature on the burden of CDI [1,2]. The absence of a significant difference in in-hospital mortality may reflect the small number of deaths in the CDI group (*n* = 1), prompt recognition and treatment of CDI with oral vancomycin (the first-line agent used in this cohort) in accordance with contemporary ESCMID and IDSA/SHEA guidance [27,28], and possible survivor bias whereby patients had to survive long enough to be diagnosed.

Corticosteroid exposure was essentially universal in both groups (>93%), in keeping with the post-RECOVERY-trial standardisation of dexamethasone therapy in hospitalised COVID-19 patients with oxygen requirement [12]. This uniform exposure may have obscured any independent contribution of corticosteroids to CDI risk in our cohort and reinforces the central role of antibiotics as the modifiable driver of CDI in this setting.

Our study period spanned five years and therefore captured successive epidemiological and therapeutic eras of the pandemic—the ancestral/Alpha-dominant first phase, the Delta wave, and the later Omicron-dominant and post-acute phases—across which admission thresholds, vaccination coverage, corticosteroid and antiviral protocols, and empirical antibiotic practices all evolved. These shifts are not merely a methodological footnote: prior work has shown that both patient profiles and the prognostic weight of individual predictors change substantially between waves. In a single-centre comparison of older COVID-19 inpatients, Siniscalchi et al. reported that the Clinical Frailty Scale failed to stratify mortality risk in unvaccinated first-wave patients yet became an independent predictor of in-hospital death in vaccinated fourth-wave patients, while baseline comorbidity burden, computed-tomography severity, and inflammatory markers differed markedly between the two phases [23]. By analogy, the drivers of CDI in our cohort were probably weighted differently across phases: broad-spectrum empirical prescribing and corticosteroid exposure are likely to have been most intense during the pre-vaccination, pre-stewardship early waves, whereas later Omicron-era admissions occurred against a background of higher vaccination coverage, attenuated disease severity, and progressively more restrictive antibiotic policies. Because the limited number of CDI events (*n* = 24) precluded a statistically meaningful wave-stratified analysis, the pooled estimates reported here should be interpreted as an average across heterogeneous pandemic phases rather than as a phase-invariant effect, and phase-specific quantification represents an important avenue for larger multicentre studies.

**Figure 4:**
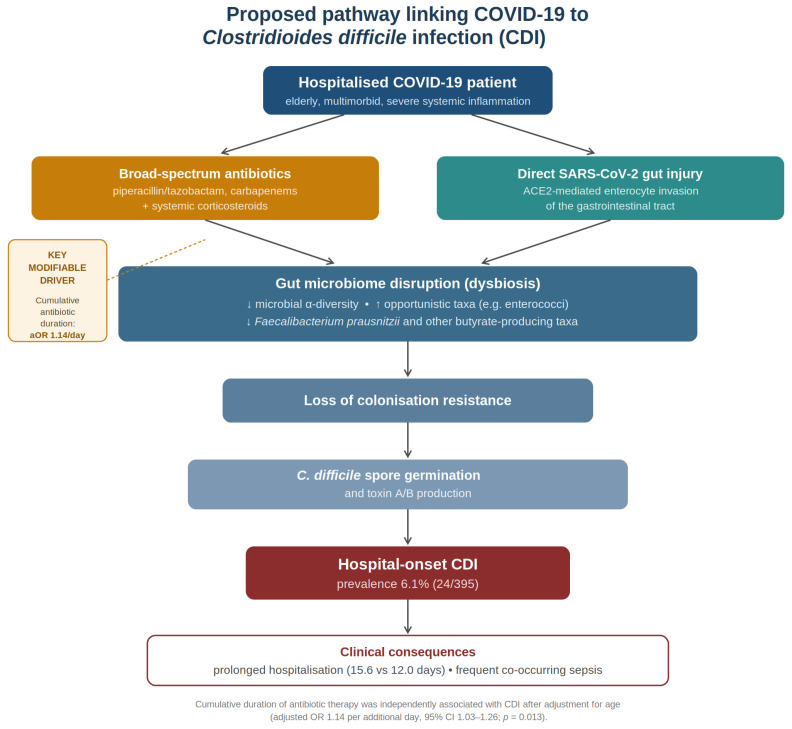
Proposed pathophysiological pathway linking hospitalised COVID-19 to hospital-onset *Clostridioides difficile* infection (CDI). Broad-spectrum antibiotic and corticosteroid exposure, together with direct SARS-CoV-2 gastrointestinal injury, converge on gut microbiome disruption (reduced α-diversity and depletion of butyrate-producing taxa such as *Faecalibacterium prausnitzii*), leading to loss of colonisation resistance, *C. difficile* spore germination and toxin production, and hospital-onset CDI (prevalence 6.1%). Cumulative duration of antibiotic therapy was the principal modifiable driver identified in this cohort (adjusted OR 1.14 per additional day, 95% CI 1.03–1.26; *p* = 0.013). Solid arrows indicate the proposed direction and sequence of the pathophysiological pathway, whereas the dashed orange arrow highlights cumulative antibiotic exposure as the principal modifiable driver identified in the present cohort.

The proposed pathophysiological pathway linking hospitalised COVID-19, antibiotic exposure, gut dysbiosis, and the development of hospital-onset CDI is summarised in Figure 4.

### Strengths and Limitations

The principal strengths of this study are the use of a consecutive, well-characterised tertiary-centre cohort spanning the entire pandemic period and the post-acute phase; the application of explicit hospital-onset CDI case definitions in line with ESCMID guidance; the consistent two-step laboratory diagnostic algorithm (GDH + toxin A/B EIA with PCR confirmation for discordant cases); the reporting of all binary comparisons with exact 95% confidence intervals using a Haldane–Anscombe correction where appropriate; the use of a deliberately parsimonious adjusted analysis respecting events-per-variable constraints; and the explicit temporal analysis of the CDI–sepsis association.

This study also has several limitations that must be acknowledged. First, the retrospective single-centre design limits generalisability and precludes formal causal inference. Second, the relatively small number of CDI cases (*n* = 24) limited statistical power, particularly for cardiovascular subgroup analyses, and constrained the multivariable model to two covariates; the absence of a statistically significant heart-failure–CDI association should therefore be interpreted as “absence of evidence at this sample size” rather than evidence of absence. Third, the long study interval encompassed distinct pandemic phases, with evolving SARS-CoV-2 variants, vaccination coverage, therapeutic protocols, corticosteroid use, antibiotic prescribing patterns, and infection-control practices, all of which may have influenced CDI ascertainment and outcomes. Fourth, CDI diagnosis was based on clinical documentation and the two-step laboratory algorithm; ribotyping data, toxin burden, and formal CDI severity scores were not systematically available, and CDI recurrence was captured only during the index hospitalisation rather than across post-discharge follow-up. Fifth, detailed regimen-level antibiotic exposure was not uniformly recorded in the non-CDI group, precluding antibiotic class-specific comparative risk estimation, including for fluoroquinolones, clindamycin, and third- or fourth-generation cephalosporins. Sixth, detailed organism-level blood-culture and antimicrobial-susceptibility data were available only for the CDI subgroup; consequently, between-group comparisons of bloodstream pathogen distribution and resistance phenotypes could not be performed. Finally, although sepsis was analysed as an important clinical outcome and its temporal relationship to CDI confirmation was characterised, residual immortal-time bias and confounding by indication cannot be fully excluded

## 5. Conclusions

In this retrospective cohort of 395 hospitalised COVID-19 patients managed at a Romanian tertiary infectious diseases centre between 2020 and 2024, hospital-onset CDI occurred in 6.1% of admissions. The cumulative duration of antibiotic therapy was independently associated with CDI after adjustment for age (adjusted OR 1.14 per day, 95% CI 1.03–1.26; *p* = 0.013), supporting prolonged broad-spectrum antimicrobial pressure as the dominant measurable and potentially modifiable driver of CDI in this setting. Heart failure was not significantly associated with CDI at the present sample size; given the limited number of events, this should be regarded as absence of evidence rather than evidence of absence, and the numerically higher prevalence of ischaemic heart disease among CDI patients requires confirmation in larger multicentre studies. Because the cohort spanned heterogeneous pandemic phases, these pooled estimates are best interpreted as an average across evolving epidemiological and prescribing contexts. CDI was also associated with prolonged hospitalisation and frequent co-occurrence of documented sepsis, although the temporal pattern observed (sepsis preceding CDI in the majority of cases) suggests that CDI more often acts as a marker of an established systemic inflammatory and antibiotic-exposed trajectory than as the direct precipitant of sepsis. These findings support strengthened antibiotic stewardship, careful CDI surveillance, and targeted infection-control strategies in hospitalised COVID-19 patients.

## Figures and Tables

**Table 1:** Laboratory instruments and platforms used for the analyses performed in this study.

Instrument/Platform	Analysis/Parameter
Sysmex XN-Series automated haematology analyser (Sysmex Corp., Kobe, Japan)	Complete blood count (CBC)—white blood cell count (WBC), neutrophil and lymphocyte counts, haemoglobin, and platelets
Roche Cobas c501 clinical chemistry module (Roche Diagnostics, Mannheim, Germany)	C-reactive protein (CRP)—turbidimetry
Roche Cobas e411/e601 immunoassay analyser (Roche Diagnostics, Mannheim, Germany)	Procalcitonin (PCT)—electrochemiluminescence immunoassay (ECLIA)
Roche Cobas e601 immunoassay analyser (Roche Diagnostics, Mannheim, Germany)	Interleukin-6 (IL-6)—ECLIA
Roche Cobas e411/e601 immunoassay analyser (Roche Diagnostics, Mannheim, Germany)	Ferritin—ECLIA
STA Compact automated coagulation analyser (Diagnostica Stago S.A.S., Asnières-sur-Seine, France) or Sysmex CS-series automated coagulation analyser (Sysmex Corporation, Kobe, Japan).	D-dimer, fibrinogen, PT/INR, aPTT—coagulometric/immunoturbidimetric
Roche Cobas c501 clinical chemistry module (Roche Diagnostics, Mannheim, Germany)	Liver and kidney function—alanine aminotransferase (ALT), aspartate aminotransferase (AST), urea, and creatinine—enzymatic/kinetic methods
Radiometer ABL-series blood gas analyser (Radiometer Medical ApS, Brønshøj, Denmark) (point-of-care)	Arterial blood gas analysis—peripheral oxygen saturation (SpO_2_), partial pressure of oxygen (pO_2_), partial pressure of carbon dioxide (pCO_2_), and lactate
QuantStudio 5 Real-Time PCR System (Thermo Fisher Scientific, Waltham, MA, USA) or Applied Biosystems 7500 Real-Time PCR System (Applied Biosystems, Foster City, CA, USA), with CE-IVD SARS-CoV-2 RT-PCR kits.	SARS-CoV-2 detection—real-time RT-PCR
BACT/ALERT 3D/VIRTUO automated blood culture system (bioMérieux, Marcy-l’Étoile, France), with FA Plus/FN Plus bottles	Blood cultures—continuous monitoring
MALDI-TOF mass spectrometry using the MALDI Biotyper system (Bruker Daltonics GmbH & Co. KG, Bremen, Germany) or the VITEK MS system (bioMérieux SA, Marcy-l’Étoile, France).	Microbial identification of positive cultures
VITEK 2 Compact automated antimicrobial susceptibility testing system (bioMérieux SA, Marcy-l’Étoile, France), with interpretation according to EUCAST clinical breakpoints.	Antimicrobial susceptibility testing
Glutamate dehydrogenase (GDH) + toxin A/B enzyme immunoassay (EIA), with PCR confirmation in discordant cases (in-house validated NAAT)	*Clostridioides difficile* diagnosis—two-step algorithm
Hospital digital radiology and multi-slice CT (≥16-slice), reported by board-certified radiologists	Chest imaging—chest X-ray and thoracic computed tomography (CT)

Abbreviations: CBC, complete blood count; WBC, white blood cell count; CRP, C-reactive protein; PCT, procalcitonin; ECLIA, electrochemiluminescence immunoassay; IL-6, interleukin-6; PT/INR, prothrombin time/international normalised ratio; aPTT, activated partial thromboplastin time; ALT, alanine aminotransferase; AST, aspartate aminotransferase; SpO_2_, peripheral oxygen saturation; pO_2_, partial pressure of oxygen; pCO_2_, partial pressure of carbon dioxide; SARS-CoV-2, severe acute respiratory syndrome coronavirus 2; RT-PCR, reverse transcription polymerase chain reaction; CE-IVD, CE-marked in vitro diagnostic; MALDI-TOF MS, matrix-assisted laser desorption/ionisation time-of-flight mass spectrometry; EUCAST, European Committee on Antimicrobial Susceptibility Testing; GDH, glutamate dehydrogenase; EIA, enzyme immunoassay; PCR, polymerase chain reaction; NAAT, nucleic acid amplification test; CT, computed tomography.

**Table 2:** Baseline characteristics, treatment exposures, and clinical outcomes according to CDI status, with unadjusted odds ratios and 95% confidence intervals.

*p*-Value	OR (95% CI)	Non-CDI (*n* = 371)	CDI (*n* = 24)	Variable
Demographic characteristics				
0.817	—	71.7 ± 11.4	71.1 ± 10.2	Age, years (mean ± SD)
0.833	1.18 (0.51–2.74)	170 (45.8)	12 (50.0)	Female sex, *n* (%)
0.224	—	28.1 ± 5.2	26.8 ± 4.9	BMI, kg/m^2^ (mean ± SD)
0.383	0.69 (0.30–1.61)	248 (66.8)	14 (58.3)	Vaccinated (≥1 dose), *n* (%)
Cardiovascular comorbidities				
0.813	1.13 (0.46–2.81)	99 (26.7)	7 (29.2)	Heart failure (any NYHA), *n* (%)
0.101	2.27 (0.93–5.52)	67 (18.1)	8 (33.3)	Ischaemic heart disease, *n* (%)
0.78	0.67 (0.19–2.32)	65 (17.5)	3 (12.5)	Atrial fibrillation, *n* (%)
0.222	0.44 (0.13–1.51)	91 (24.5)	3 (12.5)	Prior ischaemic stroke, *n* (%)
Other comorbidities				
0.78	0.74 (0.21–2.56)	60 (16.2)	3 (12.5)	Type 2 diabetes mellitus, *n* (%)
0.344	0.27 (0.04–2.06)	51 (13.7)	1 (4.2)	COPD, *n* (%)
0.248	0.20 (0.01–3.36) †	34 (9.2)	0 (0.0)	Chronic kidney disease, *n* (%)
Treatment exposures				
0.004 *	9.57 (1.28–71.74)	262 (70.6)	23 (95.8)	Antibiotic therapy, *n* (%) ‡
<0.001 *	—	5.2 ± 3.7	8.1 ± 2.2	Duration of antibiotic therapy, days (mean ± SD) ‡
1	1.59 (0.21–12.29)	347 (93.5)	23 (95.8)	Corticosteroid therapy, *n* (%)
0.145	—	7.5 ± 4.6	8.9 ± 3.8	Corticosteroid duration, days (mean ± SD)
0.337	3.42 (0.45–25.89)	323 (87.1)	23 (95.8)	Oxygen therapy, *n* (%)
Clinical outcomes				
<0.001 *	126.70 (16.78–956.98)	57 (15.4)	23 (95.8)	Sepsis (any timing), *n* (%) ‡
1	1.11 (0.06–20.30) †	6 (1.6)	0 (0.0)	Septic shock, *n* (%)
0.37	1.68 (0.37–7.70)	19 (5.1)	2 (8.3)	ICU admission, *n* (%)
0.038 *	—	12.0 ± 8.1	15.6 ± 8.2	Length of hospital stay, days (mean ± SD) ‡
1	0.53 (0.07–4.09)	28 (7.5)	1 (4.2)	In-hospital mortality, *n* (%)

Abbreviations: CDI, *Clostridioides difficile* infection; BMI, body mass index; NYHA, New York Heart Association; COPD, chronic obstructive pulmonary disease; ICU, intensive care unit; SD, standard deviation; OR, odds ratio; CI, confidence interval. * Statistically significant (*p* < 0.05); ‡ variables retained for the multivariable model and/or for figure visualisation; † Haldane–Anscombe continuity correction applied because of a zero cell.

**Table 3:** Broad-spectrum systemic antibiotic regimens administered before the onset of *Clostridioides difficile* infection in 23 of the 24 CDI patients. These regimens represent antecedent systemic antibiotic exposure for suspected or documented bacterial superinfection and were not used as CDI-directed treatment. All 24 CDI patients received oral vancomycin for CDI, as described in Section 3.3.

*n* (%)	Antibiotic Regimen
15 (65.2)	Piperacillin/tazobactam
3 (13.0)	Imipenem/cilastatin
2 (8.7)	Meropenem + linezolid
2 (8.7)	Meropenem + vancomycin
1 (4.3)	Colistin + moxifloxacin

Abbreviations: CDI, *Clostridioides difficile* infection.

**Table 4:** Blood-culture results, bloodstream pathogens identified by MALDI-TOF, and key antimicrobial-resistance phenotypes (VITEK 2, EUCAST breakpoints) in the CDI group.

Blood-Culture Parameter	*n* (%)
CDI patients with ≥1 blood-culture set obtained (of 24)	22 (91.7)
Clinically significant positive blood culture (of 22 cultured)	9 (40.9)
Sterile blood culture	12 (54.5)
Probable contaminant (single-bottle coagulase-negative staphylococcus)	1 (4.5)
Clinically significant bloodstream isolates (*n* = 9)	—
*Escherichia coli* (1 ESBL-producing)	3
*Klebsiella pneumoniae* (2 ESBL-producing; 0 carbapenemase)	3
*Enterococcus faecium* (vancomycin- and linezolid-susceptible)	2
*Pseudomonas aeruginosa* (meropenem- and ceftazidime-susceptible)	1
Sepsis microbiologically confirmed by positive blood culture (of 23 CDI patients with sepsis)	9 (39.1)

Abbreviations: CDI, *Clostridioides difficile* infection; ESBL, extended-spectrum β-lactamase. Percentages for pathogen rows are of the 9 clinically significant positive blood cultures; identification by MALDI-TOF mass spectrometry, susceptibility by VITEK 2 with EUCAST breakpoints. *C. difficile* is diagnosed by the stool two-step algorithm, not by blood culture, and is not represented among bloodstream isolates.

## Data Availability

Deidentified clinical, laboratory, and imaging data supporting the findings of this study are available from the corresponding authors upon reasonable request, subject to institutional data-sharing policies.

## Abbreviations

The following abbreviations are used in this manuscript:

ACE2	Angiotensin-converting enzyme 2
ALT	Alanine aminotransferase
aOR	Adjusted odds ratio
aPTT	Activated partial thromboplastin time
AST	Aspartate aminotransferase
BMI	Body mass index
CBC	Complete blood count
CDI	*Clostridioides difficile* infection
CI	Confidence interval
COPD	Chronic obstructive pulmonary disease
COVID-19	Coronavirus disease 2019
CRP	C-reactive protein
CT	Computed tomography
ECLIA	Electrochemiluminescence immunoassay
EIA	Enzyme immunoassay
ESCMID	European Society of Clinical Microbiology and Infectious Diseases
EUCAST	European Committee on Antimicrobial Susceptibility Testing
GDH	Glutamate dehydrogenase
ICU	Intensive care unit
IDSA	Infectious Diseases Society of America
IL-6	Interleukin-6
IQR	Interquartile range
MALDI-TOF MS	Matrix-assisted laser desorption/ionization time-of-flight mass spectrometry
NAAT	Nucleic acid amplification test
NT-proBNP	N-terminal pro–B-type natriuretic peptide
NYHA	New York Heart Association
OR	Odds ratio
ORF1ab	Open reading frame 1ab
PCR	Polymerase chain reaction
PCT	Procalcitonin
pCO_2_	Partial pressure of carbon dioxide
pO_2_	Partial pressure of oxygen
PT/INR	Prothrombin time/international normalised ratio
RT-PCR	Reverse transcription polymerase chain reaction
SARS-CoV-2	Severe acute respiratory syndrome coronavirus 2
SD	Standard deviation
SHEA	Society for Healthcare Epidemiology of America
SpO_2_	Peripheral oxygen saturation
STROBE	Strengthening the Reporting of Observational Studies in Epidemiology
WBC	White blood cell count
WHO	World Health Organization
